# Curcumin Prevents Formation of Polyglutamine Aggregates by Inhibiting Vps36, a Component of the ESCRT-II Complex

**DOI:** 10.1371/journal.pone.0042923

**Published:** 2012-08-07

**Authors:** Meenakshi Verma, Abhishek Sharma, Swarna Naidu, Ankan Kumar Bhadra, Ritushree Kukreti, Vibha Taneja

**Affiliations:** 1 Genomics and Molecular Medicine, Institute of Genomics and Integrative Biology (CSIR), Mall Road, Delhi, India; 2 Faculty of Chemistry and Biochemistry, Ruhr Universitat, Bochum, Germany; 3 Department of Research, Sir Ganga Ram Hospital, Delhi, India; 4 Department of Biotechnology, National Institute of Pharmaceutical Education and Research, S.A.S. Nagar, Punjab, India; University of Hyderabad, India

## Abstract

Small molecules with antioxidative properties have been implicated in amyloid disorders. Curcumin is the active ingredient present in turmeric and known for several biological and medicinal effects. Adequate evidence substantiates the importance of curcumin in Alzheimer's disease and recent evidence suggests its role in Prion and Parkinson's disease. However, contradictory effects have been suggested for Huntington's disease. This difference provided a compelling reason to investigate the effect of curcumin on glutamine-rich (Q-rich) and non-glutamine-rich (non Q-rich) amyloid aggregates in the well established yeast model system. Curcumin significantly inhibited the formation of htt72Q-GFP (a Q-rich) and Het-s-GFP (a non Q-rich) aggregates in yeast. We show that curcumin prevents htt72Q-GFP aggregation by down regulating Vps36, a component of the ESCRT-II (Endosomal sorting complex required for transport). Moreover, curcumin disrupted the htt72Q-GFP aggregates that were pre-formed in yeast and cured the yeast prion, [*PSI* ^+^].

## Introduction

Amyloidosis is a group of protein misfolding disorders, characterized by abnormal accumulation of insoluble fibrous protein aggregates [1]. Broadly, these amyloid proteins can be categorized as glutamine-rich (Q-rich) and non glutamine-rich (non Q-rich). Huntington's disease (HD) and Spinocerebellar ataxia [2] have been associated with expansion of Q-rich repeats. Other amyloid proteins including human prion protein (PrP), Aβ (Alzheimer disease), α-synuclein (Parkinson's disease) and Transthyretin (Transthyretin amyloidosis) lack these Q-rich regions. These amyloid proteins exhibit little or no sequence homology and are associated with a distinct clinical picture, but they share a common cross-β structure [3] suggesting a common cellular mechanism underlying the pathology of amyloid disorders.

Yeast has been well established as a successful model to decipher the molecular basis of amyloid disorders including Parkinson's disease, Alzheimer's disease, and Huntington's disease [4]–[8]. Interestingly, yeast has glutamine/asparagine (Q/N)-rich endogenous prion proteins [9] and was also shown to propagate Het-s, a non Q-rich fungal prion protein from *Podospora anserina* [10]. While a surfeit of research is focusing on uncovering the molecular mechanism of pathogenesis related to amyloidosis, several studies are now concentrating on therapeutic developments. Recently, both research and pharmaceutical industries are putting forth a huge effort to evaluate small molecules from natural products for their medicinal properties. Yeast is being exploited to screen for small molecule inhibitors with therapeutic potential for amyloid disorders [11], [12]. Furthermore, the ease of genetic manipulation and available tools in yeast allows dissection of the molecular pathways targeted by small molecules.

Curcumin, a polyphenol present in the spice turmeric, is known to have broad biological and medicinal effects including efficient anti-oxidant, anti-inflammatory and anti-proliferative activities. Overwhelming evidence now suggest that curcumin can be exploited for potential drug development for prevention and treatment of several disorders including cancer, obesity, aging, and neurodegenerative diseases. The neuroprotective effect of curcumin has been extensively studied in Aβ amyloid pathology. It binds and inhibits formation of Aβ fibrils *in vitro* [13] and reverses amyloid pathology in transgenic Alzheimer's disease (AD) animal models [14], [15]. In addition, curcumin has an inhibitory effect on the aggregation of α-synuclein [16], [17] and *in vitro* conversion of prion protein (PrP) together with the inhibition of protease-resistant PrP formation in cell culture [18]. Hence, it appeared very likely that curcumin could inhibit aggregation of expanded polyglutamine repeats in the huntingtin protein. Instead, it has previously been shown to promote aggregation of expanded polyglutamine in exon 1 of the huntingtin protein [19]. Thus, the therapeutic effect of curcumin in Huntington's disease is still questionable. In the present study, we sought to evaluate the anti-aggregation potential of curcumin for both Q-rich and non Q-rich amyloid proteins using established yeast models. Here, we find that curcumin acts through *VPS36*, which is a component of the ESCRT-II complex and prevents aggregation of exon 1 of the huntingtin protein with expanded glutamine repeats.

## Materials and Methods

### Yeast strains, Plasmids and Chemical compounds

VL2, a [*psi^−^*][*pin^−^*] and a weak [*PSI* ^+^] [*pin^−^*] strain in 74-D694 background (*ade1-14*, *ura3-52*, *leu2-3*, *112 trp1-289*, *his3-200*) was a gift from Susan Liebman. Yeast deletion strains and BY4741 were purchased from Invitrogen. The TAP (Tandem Affinity Purification)-tagged strains for *VPS36* (Cat# YSC1178-7501934) and *VPS4* (Cat# YSC1178-7503327). were procured from Open Biosystems. Deletion of *VPS36* was created in the VL2 strain by short flanking homology primers [20]. The sequences of primers used for deletion are:

Forward:ATGGAGTACTGGCATTATGTGGAAACTACGTCATCGGGCCCGAGGAGAACTTCTAGTATATC
Reverse:TCCCACTCAGTTGCTTGTCTATCAGTAAATCGCCTTCATCGTGCGTATATAGTTTCGTCTACCC

The exon 1 of human HTT gene cloned in the p426-GAL1 vector with expanded polyglutamine (htt72Q-GFP) was a gift from Susan Lindquist. The C-terminal prion domain of the Het-s gene fused with GFP is cloned under a galactose promoter in a 2 micron vector [10]. The plasmids were transformed into yeast by standard Lithium Acetate protocol [21].

Curcumin (Cat# C1386) , morin hydrate (Cat# M4008) and ascorbic acid (Cat# A4403) were all procured from Sigma. α-Tocopherol was procured from INTAS Pharmaceuticals. Curcumin and α-tocopherol were dissolved in Dimethyl sulfoxide (DMSO), morin was dissolved in methanol and ascorbic acid in sterile water.

### IC50 of compounds

The 50% inhibitory concentration (IC50) of the chemical compounds was calculated by adding different concentrations of molecules to yeast culture in early log phase (0.2 OD). The treatment was done for 14 hours at 30°C and OD_600_ was taken. The IC50 (curcumin_IC50_ = 62.5 µM, morin_IC50_ = 475 µM, α-tocopherol_IC50_ = 650 µM, ascorbic acid_IC50_ = 5 mM) was determined by plotting percentage survival versus concentration. The concentrations below IC50 for each compound were used in all the experiments.

### Quantification of amyloid aggregates in yeast by fluorescent microscopy

A [*psi^−^*][*pin^−^*] strain transformed with htt72Q-GFP or Het-s-GFP was grown in Synthetic media lacking uracil and tryptophan, respectively. Cells were then re-inoculated in Synthetic Raffinose +2% Galactose (SRaf+Gal) and grown till early log phase. Cells were transiently treated (8 hours) with different concentrations of compounds. Cell growth becomes slow during this period but the cells are then replenished with fresh inducing media and incubated for 16 hours at 30°C. After replenishment, there was no difference in growth in the treated and untreated cultures. Cells with aggregates were analyzed using a Nikon Ti-E inverted fluorescent microscope and manually counted. The percentage of cells with aggregates was calculated by counting more than 300 cells for each treatment per transformant.

To calculate the percentage of cells with htt72Q-GFP aggregates in deletion strains and BY4741 (wild type, WT), cells were grown for 48 hours in inducing media (SRaf+Gal) at 30°C and analyzed under the fluorescent microscope.

### Analysis of htt72Q-GFP aggregates by sedimentation and centrifugation assays

In the sedimentation assay, yeast cells with htt72Q-GFP aggregates were harvested and lysed using 1× lysis buffer (50 mM TrisCl, 50 mM KCl, 10 mM MgCl_2_, 5% glycerol). 150 µl of cell lysate normalized for total protein was loaded on 20% to 70% sucrose step-gradient and centrifuged for one hour at 14000 rpm. 150 µl of different fractions were carefully taken out, boiled at 95°C and run on 12% SDS-PAGE. The blot was probed with anti-GFP antibody (Cat # G6795, Sigma).

In the centrifugation assay, cell lysate normalized for total protein was centrifuged at 17,500 rpm for 1 hour at 4°C. Supernatant fraction was aspirated and the pellet was resuspended in the same volume of 1× lysis buffer. The total, supernatant and pellet fractions were resolved on 12% SDS-PAGE and analyzed by immunoblotting using anti-GFP antibody. β-actin, a housekeeping protein was used as loading control.

### Expression levels of *VPS36* and *VPS4* in the presence of curcumin

For protein levels, TAP tagged yeast strains for *VPS36* and *VPS4* were grown in rich media, re-inoculated in Synthetic complete media. Cells were treated with 20 µM and 40 µM of curcumin at 0.2 OD and incubated for 16 hours at 30°C. Protein was isolated, normalized, immunoblotted and probed with anti-TAP antibody. β-actin was used as loading control. Anti-Tap antibody was from Open Biosystems.

For mRNA levels, [*psi^−^*][*pin^−^*] cells were treated with 20 µM and 40 µM of curcumin and incubated for 16 hours at 30°C. Cells were lysed by lyticase and RNA was isolated by the Guanidinium thiocynate-phenol-chloroform extraction protocol (TRIzol method) using TRI reagent from Sigma (Cat#T9424) [22]. cDNA was prepared using High Capacity cDNA kit (Applied Biosystems) and real time PCR was carried out using SYBR Green (Fast SYBR from Applied Biosystems). *ACT1* was used as an endogenous control.

### Analyzing the effect of curcumin on yeast prion by antibiogram assay

Yeast cells contain an endogenous prion protein, Sup35, a translational termination factor. When Sup35 is functional (non-prion form), it terminates at the premature stop codon on the *ade1-14* allele. This inhibits adenine biosynthesis and results in accumulation of red pigment in the cell which gives red color to yeast cells on rich media. When Sup35 is non-functional (prion form), it no longer terminates at the stop codon on the *ade1-14* allele, adenine biosynthesis takes place and cells appear pink (weak [*PSI* ^+^]) to white in color (strong [*PSI* ^+^]) on rich media [23].

Log phase culture of a weak [*PSI* ^+^] strain was grown in YPD broth and uniformly spread on YPD plates. Plates were allowed to air dry for 30 minutes and sterile filter paper discs soaked with different concentrations of curcumin were placed on it. A sterile filter soaked with DMSO and 10 mM guanidine hydrochloride was used as negative and positive controls, respectively. These plates were incubated for 2 days at 30°C and then kept at 4°C for 2–3 days. Curing of weak [*PSI* ^+^] was monitored by change in coloration of the colonies.

## Results

### Curcumin inhibits both Q-rich (htt72Q-GFP) and non Q-rich (Het-s-GFP) aggregate formation in yeast

**Figure 1:**
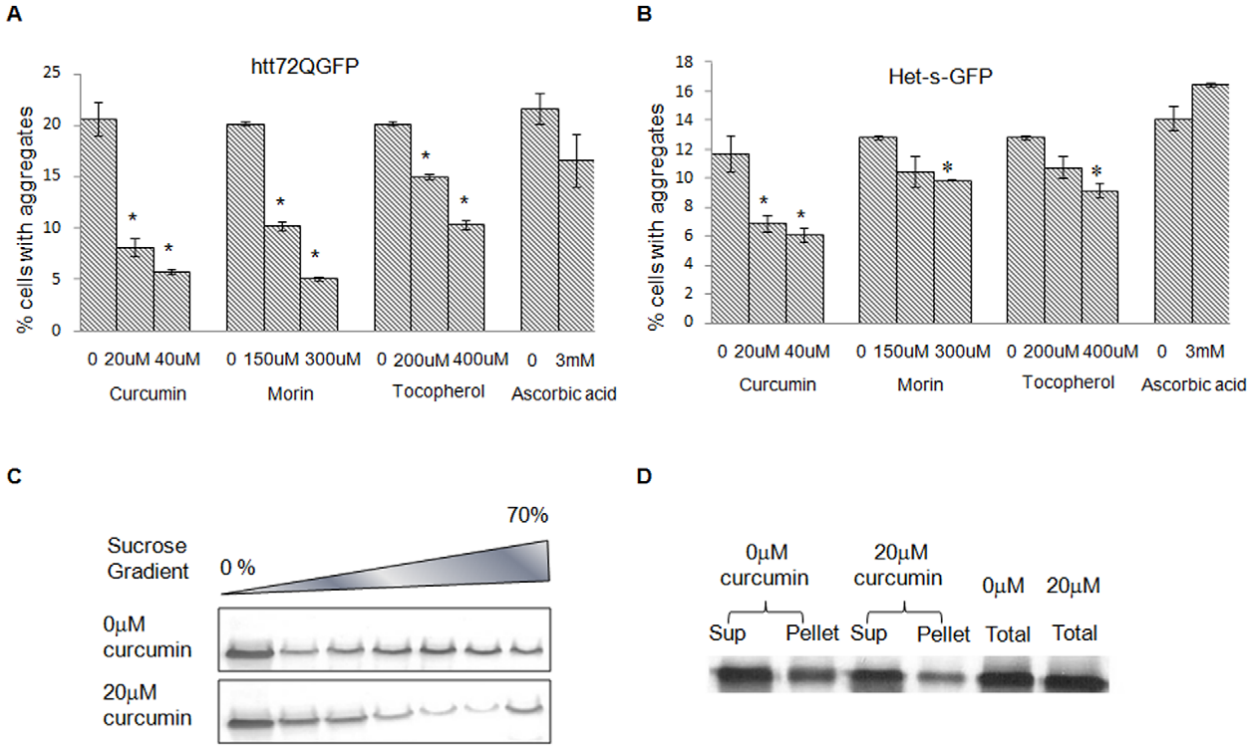
Effect of curcumin, morin, α-tochopherol and ascorbic acid on Q-rich and non Q-rich aggregates htt72Q-GFP (A) and Het-s-GFP (B) aggregation in the VL2 strain was analyzed by fluorescent microscopy. Percentage of cells with aggregates was analyzed after transient treatment with different concentrations of compounds. Aggregation counting was done for three independent transformants for each treatment. Error bars represent standard errors of the mean of triplicates. To assess the significance between treated and untreated samples, t-test was performed (* depicts *p* value<0.01). Validation of the effect of 20 µM curcumin on htt72Q-GFP by C) sucrose density gradient (0% to 70%) and D) centrifugation assay. ‘Sup’ represents the supernatant fraction. For both assays, cell lysate was normalized for total protein and processed. DMSO was used as control (0 µM) and blots were probed with anti-GFP antibody.

The inhibitory effect of curcumin has been well established for non Q-rich amyloid proteins including Alzheimer's [13] but has been shown to promote aggregation of mutant huntingtin [19]. The difference in action of curcumin on two different types of amyloid proteins prompted us to re-examine the effect of curcumin on existing yeast models for both Q-rich and non Q-rich amyloid proteins. The effect of curcumin was analyzed on htt72Q-GFP and Het-s-GFP aggregation by fluorescent microscopy in a [*psi^−^*][*pin^−^*] strain (Figure S1). More than 2-fold decrease at 20 µM and >3-fold decrease at 40 µM of curcumin were consistently observed for cells with htt72Q-GFP aggregates (Figure 1A). In case of Het-s-GFP aggregates, 1.7-fold inhibition at 20 µM and ∼2-fold at 40 µM was observed (Figure 1B). Thus, we observed a significant dose-dependent inhibitory effect on both htt72Q-GFP and Het-s-GFP aggregates.

The inhibitory effect of curcumin treatment on htt72Q-GFP aggregation was validated by sedimentation profile of htt72Q-GFP aggregates on sucrose step-gradient. Cells over expressing htt72Q-GFP were transiently (8 hours) treated with curcumin, lysed and loaded on sucrose gradient. In absence of curcumin, higher molecular weight htt72Q-GFP aggregates penetrated deeper into the higher fractions of sucrose gradient. In cells treated with 20 µM curcumin, higher molecular weight aggregates decreased significantly (fractions 4–6) and lower molecular weight aggregates increased (fractions 2 and 3) (Figure 1C). Centrifugation assay also showed a clear decrease in the pellet fraction, which again validates the inhibition of aggregation after curcumin treatment (Figure 1D).

We also analyzed the effect of three other known antioxidants on htt72Q-GFP and Het-s- GFP aggregation. Morin showed a significant dose-dependent decrease (2-fold at 150 µM and 4-fold at 300 µM) and α-tocopherol showed ∼2-fold decrease but at much higher dosage (400 µM) in htt72Q-GFP aggregates. Ascorbic acid did not show any effect on htt72Q-GFP aggregates (Figure 1A). No significant effect of morin, α-tocopherol and ascorbic acid on Het-s aggregation was observed (Figure 1B).

### Curcumin downregulates Vps36, a component of the ESCRT-II complex

**Figure 2:**
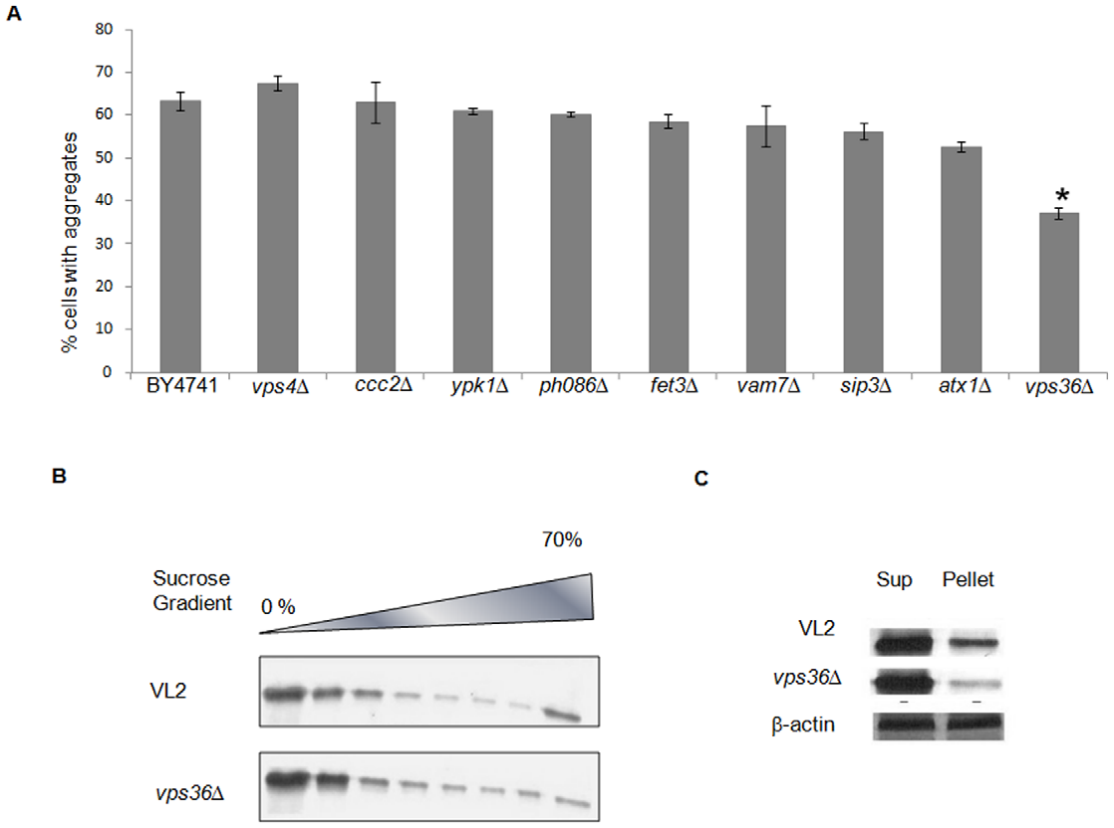
Deletion of *VPS36* decreases htt72Q-GFP aggregation induced in yeast cells. A) htt72Q-GFP aggregation was quantified in BY4741 and nine deletion strains by calculating percentage of cells with aggregates for each strain. Three independent transformants were analyzed for each strain. Only *vps36*Δ showed a significant decrease in aggregation. Error bars represent standard errors of the mean for triplicates. The significant difference in aggregation between BY4741 and *vps36*Δ was calculated by t-test (* depicts *p* value<0.05). The inhibitory effect due to *VPS36* deletion was validated by B) sucrose density gradient and C) centrifugation analysis in the VL2 background. Cells expressing htt72Q-GFP were lysed, normalized for total protein and processed. Blots were probed with anti-GFP antibody. In centrifugation assay, β-actin was used as loading control.

As curcumin exhibited an appreciable inhibition of appearance of both Q-rich and non Q-rich aggregates, we were curious to decipher the mechanism of action of curcumin. To identify the cellular target of curcumin, we utilized the information provided by the chemogenomics profiling fitness database (http://fitdb.stanford.edu/fitdb.cgi) that lists heterozygous or homozygous deletion mutants with increased sensitivity to compounds [24]. We examined htt72Q-GFP aggregation in eight homozygous yeast deletion strains (*cc2Δ*, *ypk1Δ*, *atx1Δ*, *vps36Δ*, *sip3Δ*, *pho*86*Δ*, *vam*7*Δ* and *fet3*Δ) shown to exhibit the maximum growth defect in the presence of curcumin. The growth defect in these deletion strains was confirmed in the presence of curcumin (data not shown). Out of eight, seven strains showed aggregation similar to BY4741. The *vps36*Δ strain showed a significant decrease in the percentage of cells with htt72Q-GFP aggregates (Figure 2A). We confirmed that this decrease in aggregation is not due to growth inhibition as no appreciable decrease in growth rate was observed between BY4741 and *vps36Δ*. To analyze the effect of Vps36 on htt72Q-GFP aggregation in another yeast strain background, deletion of *VPS36* was recreated in the VL2 strain and confirmed by PCR (data not shown). A similar decrease in aggregation was observed in this strain and validated by a shift towards lower fractions on sucrose density gradient (Figure 2B) and a significant decrease in pellet fraction (Figure 2C).

**Figure 3:**
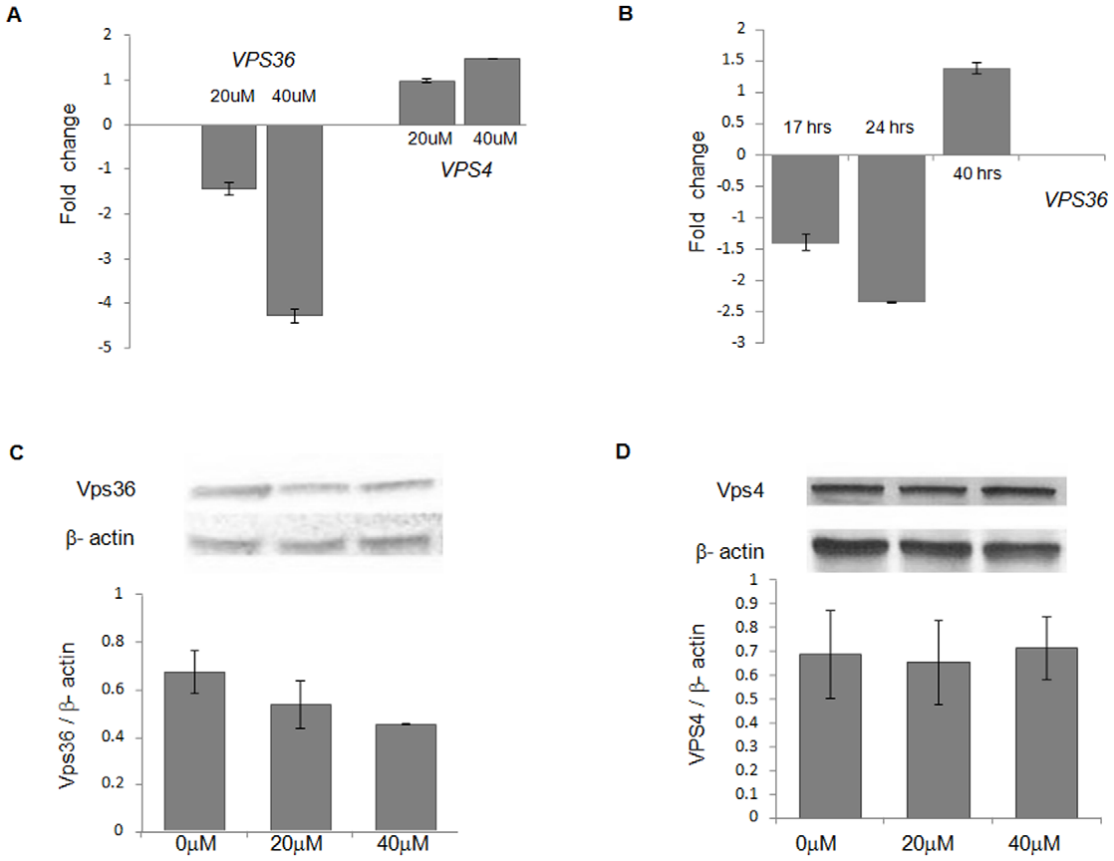
Effect of curcumin on the expression levels of Vps36 and Vps4. A) mRNA levels of Vps36 and Vps4 after 16 hours of curcumin treatment (20 µM and 40 µM) in the VL2 strain was quantified by real time PCR using SYBR green. B) Time-dependent effect of 20 µM curcumin on mRNA levels of Vps36 was determined at 17 hours, 24 hours and 40 hours in the VL2 strain. The genes were considered to be upregulated if the fold change was above 1 and downregulated if below 1. The untreated sample was normalized to 1. Each bar represents the standard error of mean of triplicates. Protein levels of C) Vps36 and D) Vps4 in respective TAP-tagged strains were determined after curcumin treatment (20 µM and 40 µM) by immunoblotting with anti-GFP antibody. The intensity of Vps36 and Vps4 (untreated and treated) protein levels was quantified by densitometer and normalized with β-actin. Error bars represent standard errors of the mean of triplicates.

*VPS36* is a component of the ESCRT-II complex and encodes Vacuolar Protein Sorting protein 36. To validate if the inhibiton of aggregation after curcumin treatment is through Vps36, the effect of curcumin on expression levels of Vps36 was analyzed. A dose and time-dependent decrease in mRNA levels of Vps36 was observed by real time PCR. VL2 strain was treated with curcumin for 16 hours, a 1.5-fold and >4-fold decrease in levels of Vps36 mRNA was observed for 20 µM and 40 µM, respectively (Figure 3A). The time-dependent effect was monitored at 20 µM curcumin after 17, 24 and 40 hours of treatment. A significant decrease in Vps36 mRNA levels at 17 hours and further decrease at 24 hours was observed. However, after 40 hours, the levels of Vps36 mRNA increased a little compared to the untreated sample (Figure 3B). A moderate decrease in the protein levels of Vps36 was also observed after curcumin treatment (Figure 3C) by probing for TAP-tagged Vps36 in BY4741 strain background. The levels of Vps4, which is involved in disassembly of ESCRT–III complex, showed no change after curcumin treatment (Figure 3A and 3D). Deletion of *vps4* did not show any change in percentage of cells with aggregation compared to BY4741 (Figure 2A). Hence, curcumin significantly downregulates Vps36 levels in yeast.

### Curcumin destabilizes Q-rich aggregates pre-formed in yeast

**Figure 4:**
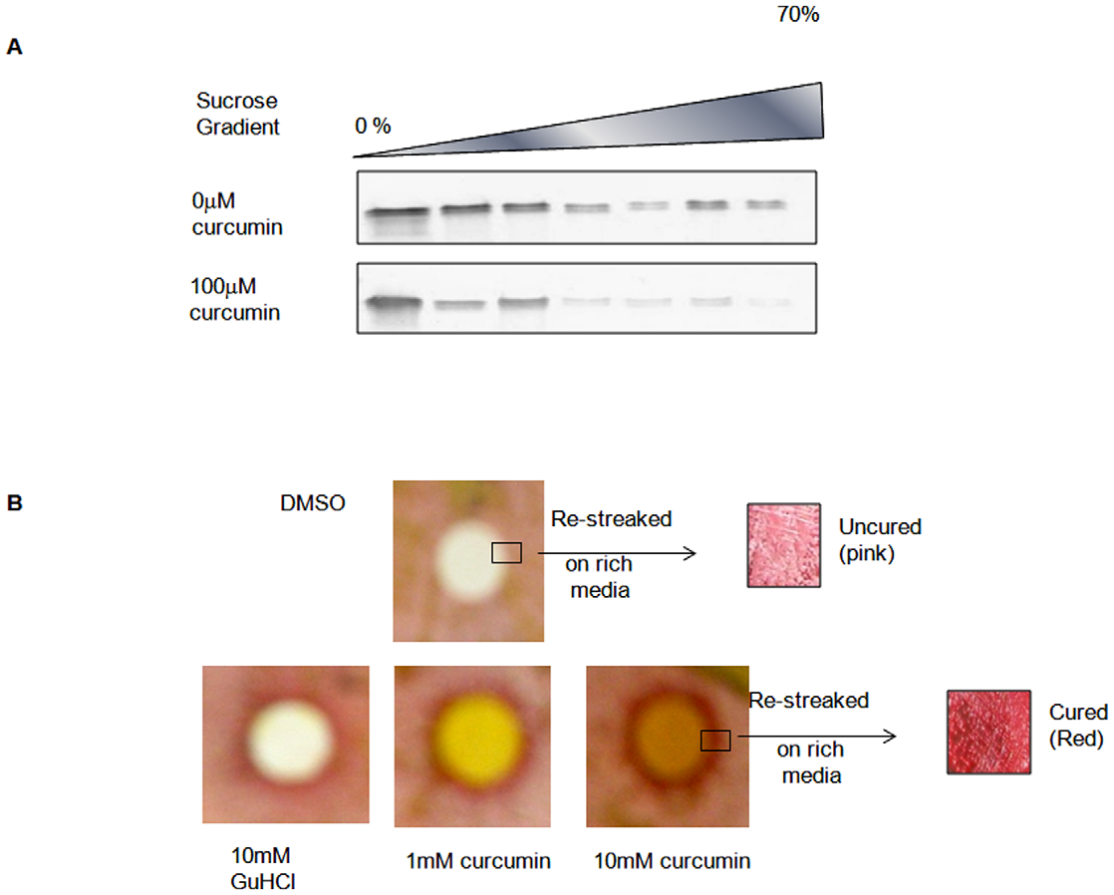
Effect of curcumin on aggregates pre-formed in yeast. A) htt72Q-GFP aggregates were pre-formed by inducing its expression in the VL2 strain. Cell lysate treated with 100 µM curcumin was subjected to sucrose density gradient and fractions were resolved on SDS-PAGE. The blot was probed using anti-GFP antibody. B) Weak [*PSI* ^+^] strain was treated with 1 mM and 10 mM of curcumin. The curing of yeast prion was observed by plating on rich media (YPD) by change in color from light pink (uncured) to red (cured). The cells near the disc were picked and restreaked on media lacking curcumin. 10 mM guanidine hydrochloride was used as a positive control and DMSO was used as a negative control.

As curcumin is known to destabilize pre-formed Aβ fibrils [25], we asked if curcumin also acts on pre-formed htt72Q-GFP aggregates. Cells with htt72Q-GFP were grown in inducing media for 48 hours, analyzed for aggregates and lysed. Cell lysate containing the pre-formed aggregates was treated *in vitro* with 100 µM curcumin at 37°C for 16 hours and analyzed by sedimentation profiling. The aggregates from untreated lysate penetrated into higher fractions of sucrose gradient, which diminished considerably after treatment (Figure 4A). The aggregates appeared more concentrated in supernatant and lower fractions of sucrose gradient. This shows that curcumin destabilizes htt72Q-GFP aggregates that are pre-formed in yeast.

The effect of curcumin was also analyzed on the endogenous yeast prion. A dose-dependent effect of curcumin on weak [*PSI* ^+^] was observed on rich media plates as an increase in red coloration of colonies around the discs soaked with increasing concentration of curcumin (Figure 4B). These red colonies were picked and restreaked on rich media lacking curcumin, which propagated as red color [*psi* ^−^] colonies as shown in figure 4B. The control discs soaked in DMSO did not cause any color change. No effect of curcumin was visible on strong [*PSI* ^+^] strain (data not shown).

## Discussion

Emerging evidence suggest a neuroprotective role of curcumin in Alzheimer's disease and several clinical trials have been initiated to examine the effect of curcumin in AD patients. Contrary to this, curcumin has been shown to increase the aggregation of mutant huntingtin and also enhance toxicity associated in PC12 cells by augmenting the proteasomal dysfunction [19]. Impairment of proteasomal machinery is implicated in other amyloid disorders including AD and HD [26]–[28], therefore, if curcumin enhances a proteasomal defect, a similar increase in aggregation is expected on treatment for other amyloid proteins. Though Aβ peptide and mutant huntingtin protein form similar cross β-sheet structures, the major difference lies in their sequence. While Aβ lacks glutamine repeats, huntingtin protein is glutamine rich. This intrigued us and we investigated aggregation of mutant huntingtin with expanded glutamine repeats and Het-s, a non Q-rich fungal prion protein, after curcumin treatment in yeast. In accordance with a protective effect of curcumin for neurodegenerative disorders, we demonstrate an inhibitory effect of curcumin on both Q-rich and non Q-rich protein aggregation in yeast and describe the likely mechanisms of inhibition.

The molecular mechanism of action of curcumin is complex and multiple molecular targets have been proposed by several independent studies [29]. Curcumin has been shown to have a therapeutic effect on AD by reducing oxidative damage [30], decreasing β-amyloid formation by inhibiting GSK-3β-mediated presenilin-1 levels [31] and Wnt/β-catenin signaling pathway activation [32] and also inhibiting trafficking and maturation of Amyloid Precursor Protein (APP) into Aβ peptide [33]. We scored aggregation in eight homozygous deletion strains hypersensitive to curcumin and only deletion of *VPS36* caused significant inhibition of formation of aggregates of mutant huntingtin. Vps36, a component of the ESCRT-II complex, appeared to be a functional target of curcumin as it was downregulated in a dose and time-dependent manner. Vps36 is involved in trafficking soluble and integral membrane proteins from the trans-golgi network to the perivacuolar region and finally to the vacuole [34]. As misfolded proteins including mutant huntingtin are known to localize in the perivacuolar region [35], we propose that *in vivo* deletion or downregulation of Vps36 caused by curcumin treatment prevents recruitment of misfolded protein to the perivacuolar compartment and thus inhibits formation of visible large aggregates. The importance of protein trafficking pathways in aggregation of amyloid proteins has been demonstrated previously. Several genes from endocytic pathway and vacuolar protein sorting are known to affect aggregation of amyloid proteins including mutant huntingtin [36] and prion proteins [37]. In compliance with previous work, our work further strengthens that protein trafficking pathways can be exploited for developing small molecule inhibitors of amyloid disorders.

Curcumin has been shown to destabilize Aβ fibrils but disassembly of mutant huntingtin by curcumin has not been described earlier. *In vitro*, disruption or remodelling of mutant huntingtin aggregates pre-formed in yeast clearly supports anti-aggregation potential of curcumin. Furthermore, our findings are supported by a very recent report that shows a clear decrease in number of aggregates in the striatum of knock-in HD mouse model [38]. Curcumin is known to cross the blood-brain barrier but bioavailibity is still poor. Hence, analogs of curcumin or combination of small molecules that increase the bioavailability of the active molecule need to be evaluated.

Morin, a polyphenol, present in red wine, has been suggested to have potential for developing therapies for AD and tauopathies by inhibiting GSK-3β [39], [40]. Recently, it has been reported to inhibit amyloid formation of Islet Amyloid Polypeptide (IAPP) [41]. We show that morin is an effective inhibitor of mutant huntingtin aggregates. Morin treatment also led to slight inhibition of non-Q rich aggregates. Further work is required to understand the mechanism of action of morin.

The key findings of our study are that curcumin is a more potent inhibitor of both Q-rich and non Q-rich aggregates than morin and α-tocopherol. Furthermore, it inhibits mutant huntingtin aggregation by acting through protein trafficking pathways and also destabilizes pre-formed aggregates. Based on our data and previous studies, curcumin is a promising compound for development of preventives and therapeutics for both Q-rich and non Q-rich amyloid disorders.

## Supporting Information

Figure S1 **Microscopic image of cells showing the effect of curcumin on htt72Q-GFP aggregates in yeast.**
(PPT)

## References

[1] Westermark P (2005) Aspects on human amyloid forms and their fibril polypeptides. FEBS J 272: 5942–5949. 16302959 10.1111/j.1742-4658.2005.05024.x

[2] Shastry BS (2003) Neurodegenerative disorders of protein aggregation. Neurochem Int 43: 1–7. 12605877 10.1016/s0197-0186(02)00196-1

[3] Nelson R, Eisenberg D (2006) Structural models of amyloid-like fibrils. Adv Protein Chem 73: 235–282. 17190616 10.1016/S0065-3233(06)73008-X

[4] Willingham S, Outeiro TF, DeVit MJ, Lindquist SL, Muchowski PJ (2003) Yeast genes that enhance the toxicity of a mutant huntingtin fragment or α-synuclein. Science 302: 1769–1772. 14657499 10.1126/science.1090389

[5] Zabrocki P, Pellens K, Vanhelmont T, Vandebroek T, Griffioen G, et al (2005) Characterization of α-synuclein aggregation and synergistic toxicity with protein tau in yeast. FEBS J 272: 1386–1400. 15752356 10.1111/j.1742-4658.2005.04571.x

[6] Boone C, Bussey H, Andrews BJ (2007) Exploring genetic interactions and networks with yeast. Nat Rev Genet 8: 437–449. 17510664 10.1038/nrg2085

[7] Treusch S, Hamamichi S, Goodman JL, Matlack KE, Chung CY, et al (2011) Functional links between Aβ toxicity, endocytic trafficking, and Alzheimer's disease risk factors in yeast. Science 334: 1241–1245. 22033521 10.1126/science.1213210 PMC3281757

[8] Sherman MY, Muchowski PJ (2003) Yeast Models as Tools to Study Neurodegenerative Disorders. NeuroMolecular Medicine 4: 133–146. 14528057 10.1385/NMM:4:1-2:133

[9] Shkundina IS, Ter-Avanesyan MD (2007) Prions. Biochemistry (Mosc) 72: 1519–1536. 18282140 10.1134/s0006297907130081

[10] Taneja V, Maddelein ML, Talarek N, Saupe SJ, Liebman SW (2007) A non-Q/N-rich prion domain of a foreign prion, [Het-s], can propagate as a prion in yeast. Mol Cell 27: 67–77. 17612491 10.1016/j.molcel.2007.05.027 PMC1995001

[11] Roberts BE, Duennwald ML, Wang H, Chung H, Lopreiato NP (2009) A synergistic small-molecule combination directly eradicates diverse prion strain structures. Nat chem Biol 5: 936–946. 19915541 10.1038/nchembio.246 PMC2909773

[12] Sarkar S, Perlstein EO, Imarisio S, Pineau S, Cordenier A (2007) Small molecules enhance autophagy and reduce toxicity in Huntington's disease models. Nat Chem Biol 3: 331–338. 17486044 10.1038/nchembio883 PMC2635561

[13] Yang F, Lim GP, Begum AN, Ubeda OJ, Simmons MR (2005) Curcumin inhibits formation of amyloid beta oligomers and fibrils, binds plaques, and reduces amyloid *in vivo* . J Biol Chem 280: 5892–6901. 15590663 10.1074/jbc.M404751200

[14] Lim GP, Chu T, Yang F, Beech W, Frautschy SA (2001) The curry spice curcumin reduces oxidative damage and amyloid pathology in an Alzheimer transgenic mouse. J Neurosci 21: 8370–8377. 11606625 10.1523/JNEUROSCI.21-21-08370.2001 PMC6762797

[15] Garcia-Alloza M, Borrelli LA, Rozkalne A, Hyman BT, Bacskai BJ (2007) Curcumin labels amyloid pathology *in vivo*, disrupts existing plaques, and partially restores distorted neurites in an Alzheimer mouse model. J Neurochem 102: 1095–1104. 17472706 10.1111/j.1471-4159.2007.04613.x

[16] Ahmad B, Lapidus L (2012) Curcumin prevents aggregation in α-synuclein by increasing reconfiguration rate. J Biol Chem 287: 9193–9199. 22267729 10.1074/jbc.M111.325548 PMC3308736

[17] Pandey N, Strider J, Nolan WC, Yan SX, Galvin JE (2008) Curcumin inhibits aggregation of alpha-synuclein. Acta Neuropathol 115: 479–489. 18189141 10.1007/s00401-007-0332-4

[18] Hafner-Bratkovic I, Gaspersic J, Smid LM, Bresjanac M, Jerala R (2008) Curcumin binds to the alpha-helical intermediate and to the amyloid form of prion protein - a new mechanism for the inhibition of PrP(Sc) accumulation. J Neurochem 104: 1553–1564. 17996023 10.1111/j.1471-4159.2007.05105.x

[19] Dikshit P, Goswami A, Mishra A, Nukina N, Jana NR (2006) Curcumin enhances the polyglutamine-expanded truncated N- terminal huntingtin-induced cell death by promoting proteasomal malfunction. Biochemical and Biophysical Research Communications 342: 1323–1328. 16516148 10.1016/j.bbrc.2006.02.104

[20] Baudin A, Ozier-Kalogeropoulos O, Denouel A, Lacroute F, Cullin C (1993) Simple and efficient method for direct gene deletion in *Saccharomyces cerevisiae* . Nucleic Acids Res 21: 3329–3330. 8341614 10.1093/nar/21.14.3329 PMC309783

[21] Gietz RD, Woods RA (2002) Transformation of yeast by lithium acetate/single-stranded carrier DNA/polyethylene glycol method. Methods in enzymology 350: 87–96. 12073338 10.1016/s0076-6879(02)50957-5

[22] Yılmaz R, Akça O, Baloglu MC, Öz MT, Avni H, et al (2012) Optimization of yeast (*Saccharomyces cerevisiae*) RNA isolation method for real-time quantitative PCR and microarray analysis. African Journal of Biotechnology 11: 1046–1053.

[23] Inge-Vechtomov SG, Tikhodeev ON, Karpova TS (1988) Selective systems for obtaining recessive ribosomal suppressors in yeast *Saccharomyces cerevisiae* . Genetika 24: 1159–1165. 3053330

[24] Giaever G, Flaherty P, Kumm J, Proctor M, Nislow C, et al (2004) Chemogenomic profiling: identifying the functional interactions of small molecules in yeast. Proc Natl Acad Sci USA 101: 793–798. 14718668 10.1073/pnas.0307490100 PMC321760

[25] Ono K, Hasegawa K, Naiki H, Yamada M (2004) Curcumin has potent anti-amyloidogenic effects for Alzheimer's beta-amyloid fibrils *in vitro* . J Neurosci Res 75: 742–750. 14994335 10.1002/jnr.20025

[26] Upadhya SC, Hegde AN (2007) Role of the ubiquitin proteasome system in Alzheimer's disease. BMC Biochemistry 8: S1–S12. 18047736 10.1186/1471-2091-8-S1-S12 PMC2106363

[27] Davies JE, Sarkar S, Rubinsztein DC (2007) The ubiquitin proteasome system in Huntington's disease and the spinocerebellar ataxias. BMC Biochemistry 8: S1–S2. 18047739 10.1186/1471-2091-8-S1-S2 PMC2106366

[28] Keller JN, Hanni KB, Markesbery WR (2000) Impaired Proteasome Function in Alzheimer's disease. J Neurochem 75: 436–439. 10854289 10.1046/j.1471-4159.2000.0750436.x

[29] Lin JK (2007) Molecular Targets of Curcumin. Adv Exp Med Biol 595: 227–43. 17569214 10.1007/978-0-387-46401-5_10

[30] Lim GP, Chu T, Yang F, Beech W, Frautschy SA, et al (2001) The Curry Spice Curcumin Reduces Oxidative Damage and Amyloid Pathology in an Alzheimer Transgenic Mouse. J Neurosci 21: 8370–8377. 11606625 10.1523/JNEUROSCI.21-21-08370.2001 PMC6762797

[31] Xiong Z, Hongmei Z, Lu S, Yu L (2011) Curcumin mediates presenilin-1 activity to reduce β-amyloid production in a model of Alzheimer's Disease. Pharmacol Rep 63: 1101–1108. 22180352 10.1016/s1734-1140(11)70629-6

[32] Zhang X, Yin WK, Shi XD, Li Y (2011) Curcumin activates Wnt/β-catenin signaling pathway through inhibiting the activity of GSK-3β in APPswe transfected SY5Y cells. Eur J Pharm Sci 42: 540–546. 21352912 10.1016/j.ejps.2011.02.009

[33] Zhang C, Browne A, Child D, Tanzi RE (2010) Curcumin decreases amyloid-beta peptide levels by attenuating the maturation of amyloid-beta precursor protein. J Biol Chem 285: 28472–28480. 20622013 10.1074/jbc.M110.133520 PMC2937872

[34] Raymond CK, Howald-Stevenson I, Vater CA, Stevens TH (1992) Morphological classification of the yeast vacuolar protein sorting mutants: evidence for a prevacuolar compartment in class E vps mutants. Mol Biol Cell 3: 1389–402. 1493335 10.1091/mbc.3.12.1389 PMC275707

[35] Kaganovich D, Kopito R, Frydman J (2006) Misfolded proteins partition between two distinct quality control compartments. Biochemical and Biophysical Research Communications 342: 1323–1328. 18756251 10.1038/nature07195 PMC2746971

[36] Meriin AB, Zhang X, Alexandrov IM, Salnikova AB, Ter-Avanesian MD, et al (2007) Endocytosis machinery is involved in aggregation of proteins with expanded polyglutamine domains. FASEB J 8: 1915–1925. 10.1096/fj.06-6878com 17341688

[37] Mathur V, Taneja V, Sun Y, Liebman SW (2010) Analyzing the birth and propagation of two distinct prions, [*PSI* ^+^] and [Het-s]_y_, in yeast. Mol Biol Cell 21: 1449–1461. 20219972 10.1091/mbc.E09-11-0927 PMC2861605

[38] Hickey MA, Zhu C, Medvedeva V, Lerner RP, Patassini S, et al (2012) Improvement of neuropathology and transcriptional deficits in CAG 140 knock-in mice supports a beneficial effect of dietary curcumin in Huntington's disease. Mol Neurodegener 7–12. 10.1186/1750-1326-7-12 PMC3348060 22475209

[39] Jagota S, Rajadas J (2012) Effect of phenolic compounds against Aβ aggregation and Aβ- induced toxicity in transgenic *C. elegans* . Neurochem Res 37: 40–48. 21858698 10.1007/s11064-011-0580-5

[40] Gong EJ, Park HR, Kim ME, Piao S, Lee E, et al (2011) Morin attenuates tau hyperphosphorylation by inhibiting GSK3β. Neurobiol Dis 4: 223–230. 10.1016/j.nbd.2011.07.005 PMC3166962 21782947

[41] Noor H, Cao P, Raleigh DP (2012) Morin hydrate inhibits amyloid formation by islet amyloid polypeptide and disaggregates amyloid fibers. Protein Sci 21: 373–382. 22238175 10.1002/pro.2023 PMC3375438

