# Supplementary material for: Curcumin Prevents Formation of Polyglutamine Aggregates by Inhibiting Vps36, a Component of the ESCRT-II Complex
Source: PLoS One. 2012 Aug 7;7(8):e42923. doi: 10.1371/journal.pone.0042923 (PMC3413662; doi:10.1371/journal.pone.0042923)
Supplement: Figure S1 — Microscopic image of cells showing the effect of curcumin on htt72Q-GFP aggregates in yeast. (PPT) [file pone.0042923.s001.ppt]

## Slide 1
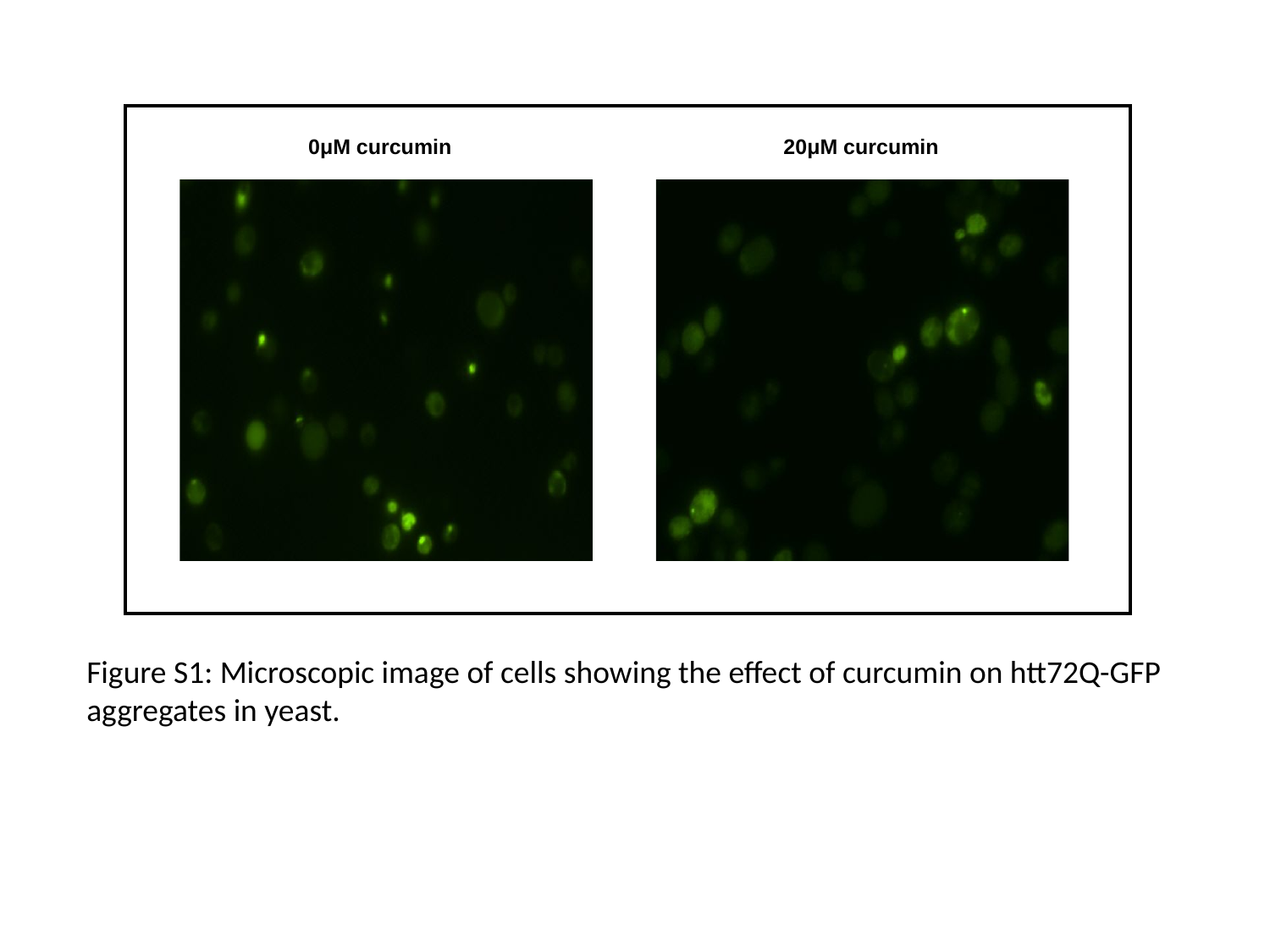

0μM curcumin
20μM curcumin
Figure S1: Microscopic image of cells showing the effect of curcumin on htt72Q-GFP aggregates in yeast.
